# Subthreshold stochastic vestibular stimulation affects balance-challenged standing and walking

**DOI:** 10.1371/journal.pone.0231334

**Published:** 2020-04-10

**Authors:** Chiara Piccolo, Amanda Bakkum, Daniel S. Marigold

**Affiliations:** Department of Biomedical Physiology and Kinesiology, Simon Fraser University, Burnaby, British Columbia, Canada; University of Rochester, UNITED STATES

## Abstract

Subthreshold stochastic vestibular stimulation (SVS) is thought to enhance vestibular sensitivity and improve balance. However, it is unclear how SVS affects standing and walking when balance is challenged, particularly when the eyes are open. It is also unclear how different methods to determine stimulation intensity influence the effects. We aimed to determine (1) whether SVS affects stability when balance is challenged during eyes-open standing and overground walking tasks, and (2) how the effects differ based on whether optimal stimulation amplitude is derived from sinusoidal or cutaneous threshold techniques. Thirteen healthy adults performed balance-unchallenged and balance-challenged standing and walking tasks with SVS (0–30 Hz zero-mean, white noise electrical stimulus) or sham stimulation. For the balance-challenged condition, participants had inflatable rubber hemispheres attached to the bottom of their shoes to reduce the control provided by moving the center of pressure under their base of support. In different blocks of trials, we set SVS intensity to either 50% of participants’ sinusoidal (motion) threshold or 80% of participants’ cutaneous threshold. SVS reduced medial-lateral trunk velocity root mean square in the balance-challenged (p < 0.05) but not in the balance-unchallenged condition during standing. Regardless of condition, SVS decreased step-width variability and marginally increased gait speed when walking with the eyes open (p < 0.05). SVS intensity had minimal effect on the standing and walking measures. Taken together, our results provide insight into the effectiveness of SVS at improving balance-challenged, eyes-open standing and walking performance in healthy adults.

## Introduction

The vestibular system is important for encoding motion of the head, which contributes to maintaining an upright posture [1,2]. Age-related vestibular hair cell loss and vestibular impairments due to injury or disease affect vestibular sensitivity, which may reduce balance and increase the likelihood of falls [3,4]. To mitigate this problem, researchers are developing novel approaches using low levels of electrical stimulation. These techniques are based on a phenomenon known as stochastic resonance (SR), whereby the addition of noise or random interference may enhance the capacity to detect a weak signal or process information [5,6]. Indeed, SR can enhance sensory perception and motor function [5–12].

Recently, several groups of researchers have demonstrated that the application of subthreshold stochastic vestibular stimulation (SVS), also known as noisy galvanic vestibular stimulation, is associated with improved balance control during standing and changes in walking patterns [13–20]. For example, SVS results in reduced postural sway length and velocity during eyes-open quiet standing in young and older adults [15,16] and during eyes-closed quiet standing in patients with bilateral vestibulopathy [13,17]. In addition, others found increased gait speed and changes in stride measures with SVS during treadmill and overground walking in healthy adults and patients with bilateral vestibulopathy [18–20]. If subthreshold SVS provides noise that increases the sensitivity of the vestibular system to respond to natural vestibular inputs, then its effectiveness should also be evident in more balance-challenged situations, when the vestibular system’s contribution to balance is presumably more important. These situations may include standing and walking on different types of terrain or dealing with perturbations due to physical contact with other people or sudden support surface changes.

Subthreshold SVS may indeed facilitate upright posture when balance is challenged. For example, some healthy adults show improved balance function in response to SVS while standing with their eyes closed on a compliant surface [21,22]. In contrast, Pal et al. [23] studied the effect of SVS on postural sway, with and without vision, while healthy adults and patients with Parkinson’s disease stood on foam. SVS had no significant overall effect on sway in healthy participants, and only a small decrease in sway in patients in the eyes-closed condition. However, these researchers used the same stimulus intensities for all participants (100 μA, 300 μA, and 500 μA, in different trials), rather than determining the optimal parameters like more recent work. It is possible that these stimulus intensities fell too much above or below a participant’s optimal level. As such, only some participants may have experienced a change in postural sway with SVS, which may partially explain the lack of effects in the eyes-open, balance-challenged condition. Thus, it is still unclear how SVS affects balance-challenged standing with the eyes open despite the fact that individuals typically stand (and walk) when visual feedback is available.

A similar paucity of studies exists for balance-challenged walking. In one such experiment, SVS decreased the variability of gait cycle timing and trunk acceleration while participants walked at a fixed speed on a laterally oscillating treadmill [24]. This supports the notion that SVS may facilitate balance-challenged walking, but it is important to recognize that many groups have found that there are significant differences between treadmill walking and more natural, overground walking. For instance, optic flow patterns are not the same, since there is little forward-backward motion while on a treadmill. In addition, joint range of motion and variability, step width and step-width variability, and metrics of local dynamic stability (quantified by maximum finite-time Lyapunov exponents) differ between these walking conditions [25–27]. Although Temple et al. [28] recently found that SVS resulted in faster completion time on a mobility course that required overground walking on foam while adapting to a visual perturbation, they did not report the effects of SVS on measures of balance or gait performance. As such, it is unclear whether the improvements in stability reported in Mulavara et al. [24] translate to overground walking. Therefore, in the present study, we aimed to determine whether and how SVS affects performance when balance is challenged during eyes-open standing and overground walking tasks. We hypothesized that SVS, compared to sham stimulation, would reduce trunk motion and measures of variability in both tasks, particularly in the balance-challenged condition.

Unfortunately, there is substantial variation in the methods used to assess the efficacy of SVS on balance control, making the identification of best practices and direct comparisons between studies problematic. In particular, there is no universal method used to determine the optimal vestibular stimulation amplitude for individual participants. Two threshold techniques are commonly used in SVS studies: sinusoidal and cutaneous. For the sinusoidal technique, a 1 Hz sinusoidal waveform with currents ranging from 0–1500 μA is applied to determine a motion threshold [21,24]; that is, the lowest stimulation intensity that elicits perceived or observable sway of the head or body. Alternatively, for the cutaneous technique, varying stimulation intensities using a 0–30 Hz stochastic signal are applied in a stepwise manner until a sensation is felt on the skin under the electrode site [17–20]. Both sinusoidal and cutaneous threshold techniques have proved beneficial, though, as we show in this study, yield different stimulation intensities. However, because no studies have directly compared these techniques, it is not known whether one is more effective than the other or if they prompt similar responses. Therefore, we also aimed to determine how the effects of SVS differ based on whether the optimal stimulation amplitude is derived from sinusoidal or cutaneous threshold techniques. As such, this study represents an initial attempt to provide recommendations for future work and improve consistency among studies.

## Materials and methods

### Participants

Thirteen adults (age: 22.3 ± 2.8 years; 7 males, 6 females) with no recent history of head trauma (within the last year), neurological or musculoskeletal disorders, and no metal or electronic implants anywhere in their body, participated in this study. The Office of Research Ethics at Simon Fraser University approved the study (#2018s0046), and all participants gave informed, written consent before participating in the experiment.

### Protocol

All participants performed balance-unchallenged and balance-challenged standing and walking tasks with SVS (under two different intensities depending on the condition) or with sham stimulation. Regardless of balance condition, a safety harness system attached to the participants served to prevent falling to the ground in the event the participant lost balance.

**Fig 1:**
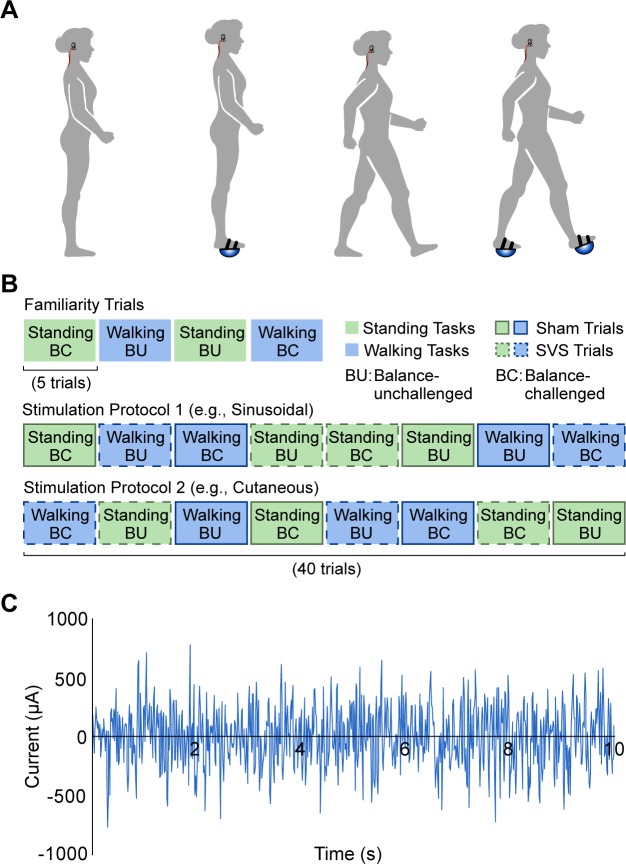
Experimental details. (A) Illustrations of the different conditions: standing with and without the balance-challenging inflatable rubber hemispheres attached to the shoes; walking with and without the balance-challenging inflatable rubber hemispheres attached to the shoes. (B) An example of the experimental protocol for one participant. We randomized the blocks of trials within each stimulation protocol. (C) An example of the zero mean, 0–30 Hz white-noise stimulation applied in the stochastic vestibular stimulation (SVS) blocks of trials.

For the standing tasks, we instructed participants to stand with their arms at their sides, their feet as close together as possible without touching, and their gaze focused directly in front of them. For the balance-unchallenged condition, participants performed this task with comfortable walking shoes. For the balance-challenged condition, participants performed the standing task with inflatable rubber hemispheres (radii: 8.5 cm) attached to the soles of their shoes to reduce the control afforded by shifting the center of pressure under the base of support (Fig 1A). We have previously shown this manipulation greatly challenges balance [29]. We ensured that the rubber hemispheres were fully inflated (0.5–1 psi) prior to testing each participant. Due to the width of the rubber hemispheres, the medial-lateral distance between the participants feet was greater (~10 cm) in the balance-challenged condition compared to the balance-unchallenged condition. However, we were interested in the effects of SVS and threshold techniques and not whether balance was different between conditions. Trials lasted five seconds. We chose this duration because we found in pilot experiments that it was very difficult to stand on the rubber hemispheres in the balance-challenged condition for long durations.

For the walking tasks, participants walked at least six steps along a straight path in the lab with their gaze focused directly ahead. We instructed participants to walk at a self-selected pace, starting with their right foot. For the balance-unchallenged condition, participants performed the walking task in comfortable walking shoes. For the balance-challenged condition, participants performed the walking task with the abovementioned inflatable rubber hemispheres attached to the soles of their shoes (Fig 1A).

Participants first performed twenty trials (five per task and balance condition) prior to the experimental trials to familiarize themselves with the protocol. Next, participants performed both tasks, with either SVS or sham stimulation, and with and without challenged balance, in blocks of five trials each (for a total of 40 experimental trials, excluding familiarization trials). This is illustrated in Fig 1B. We randomized the order of these blocks of trials for each participant. During the standing task, if a participant took a step, engaged the safety harness, or was helped by a researcher standing adjacent (thus, indicating a loss of balance), we marked the trial as incomplete and repeated it. We delivered SVS (or sham stimulation) starting three seconds before the beginning of each trial for a total duration of 10 seconds. Applying SVS for short durations similar to this can reduce sway in healthy young adults during eyes-open standing on normal ground [16]. We repeated this protocol for each of the two stimulation intensity conditions (for a total of 80 experimental trials). We counterbalanced the orders of the SVS intensities across participants.

An Optotrak Certus motion capture camera (Northern Digital, Waterloo, ON, Canada), placed perpendicular to the walkway, recorded kinematic data at 120 Hz from infrared-emitting position markers placed on the participant’s head (located at the temple), chest (over the manubrium of the sternum), and bilaterally on the heel, mid-foot (second-third metatarsal head), fifth metatarsal, and toe (third metatarsal).

### Stochastic vestibular stimulation

We used a battery-driven linear stimulus isolator (A395D, World Precision Instruments, Sarosta, FL, USA) to deliver the SVS through square (5 x 5 cm) electrodes (Axelgaard Manufacturing, Fallbrook, CA, USA) placed over the mastoid processes. We cleaned and dried the skin under the electrode site and applied a layer of Signa Gel electrode gel (Parker Laboratories Corp., Fairfield, NJ, USA) to each electrode before positioning them on the skin surface. Additionally, we applied sports pre-wrap around the head of the participant to secure the electrodes and ensure a uniform current distribution. We developed the SVS signal, which consisted of zero mean, Gaussian-distributed white noise within a frequency range of 0–30 Hz [19–22,24], using a custom LabVIEW program (National Instruments, Austin, TX). An example of the stimulus is shown in Fig 1C. Electrical vestibular stimulation activates the afferents of both semicircular canals and otolith organs [30–32].

We used two different techniques to determine the intensity of SVS for each individual participant: motion perception due to sinusoidal stimulation and cutaneous perception of a stochastic stimulus. This produced two different stimulation intensities, which we used in different experimental conditions (Fig 1B). For both techniques, participants sat on a backless stool facing forward, with their eyes closed and their feet placed flat on the ground.

For the sinusoidal technique, we applied a 1 Hz sinusoidal electrical signal to determine the motion threshold [21,24]. We instructed participants to report any perceived head motion by raising their right hand for the duration of the sensation. We delivered an initial 1500 μA peak current to allow the participant to recognize the type of motion sensation they should expect. Thereafter, the stimulus consisted of a sinusoidal waveform over a 10 second period. We used peak amplitudes of 50, 100, 150, 200, 250, 300, 350, 400, 450, 500, 550, 600, 650, 700, 750, 800, 850, 900, 950, 1000, 1100, and 1200 μA. We used 100 μA increments for the higher amplitudes because, based on past research, we did not expect to see thresholds in this range, and we wanted to reduce the number of stimulation trials. We randomized the order of each stimulation level and included a rest interval (up to 30 seconds) between each stimulation attempt. We classified the sinusoidal threshold as the lowest current level where the participant perceived head motion.

For the cutaneous technique, we applied the same 0–30 Hz stimulation that we used during the experimental trials. The initial stimulation started at 50 μA and increased by 25 μA until the participant indicated a cutaneous sensation (i.e., tingling under the electrodes). We included a rest interval (up to 30 seconds) between each stimulation attempt and repeated this process twice to determine the lowest current level that induced a cutaneous sensation.

For the sinusoidal-based condition, we set the SVS intensity to 50% of the participant’s sinusoidal threshold [21,28]. For the cutaneous-based condition, we set the SVS intensity to 80% of the participant’s cutaneous threshold [17,19,20]. We chose the specific threshold percentages because these studies have used them and found them to be optimal at improving balance. The sinusoidal threshold technique (304 ± 81 μA) resulted in significantly greater stimulation amplitudes (two-tailed paired t test: t_12_ = -3.36, p = 0.006) in comparison to the cutaneous threshold technique (249 ± 84 μA), as illustrated in Table 1. We included sham stimulation to serve as a control for each task and balance condition. Specifically, the sham waveform consisted of a gradual ramp increase over one second to the relevant intensity used in the SVS conditions (based on the thresholds), followed by a gradual decrease to zero. This form of sham stimulation is commonly employed in non-invasive brain stimulation studies (see, for example, [33]).

**Table 1:** Stimulation thresholds.

	Threshold Amplitude (μA)		Stimulation Amplitude (μA)	
Participant	Sinusoidal	Cutaneous	Sinusoidal*	Cutaneous**
1	750	375	375	300
2	800	375	400	300
3	650	300	325	240
4	800	375	400	300
5	400	150	200	120
6	500	175	250	140
7	700	350	350	280
8	700	400	350	320
9	550	325	275	260
10	250	150	125	120
11	550	475	275	380
12	550	225	275	180
13	700	375	350	300
**Mean (SD)**	**608 (162)**	**312 (104)**	**304 (81)**	**249 (84)**

* 50% of threshold.

** 80% of threshold.

### Data and statistical analysis

We analyzed kinematic data (filtered using a fourth-order, 6 Hz low-pass Butterworth algorithm) to calculate displacement and velocity profiles of select position markers. We calculated the measures over the entire trial for the standing tasks and over four heel strikes (i.e., three consecutive steps or one full stride of each leg) in the middle of the walkway for the walking tasks. For the walking task, this represented the middle steps, which thus avoids the initial acceleration to start walking and the deceleration at the end. To assess stability in both tasks, we calculated the medial-lateral (ML) and anterior-posterior (AP) trunk velocity root mean square (RMS) using the chest marker. For the standing tasks, we calculated the average minimum distance of the center of the trunk to the edge of the base of support. In the AP direction, this represented the difference between the chest marker and a toe marker on the right foot. In the ML direction, this represented the difference between the chest marker and a marker on the fifth metatarsal of the right foot. For the walking tasks, we also calculated gait speed over a distance of three meters based on the time it took for the chest marker to move this amount. Furthermore, we calculated step width using the ML distance between the moment of foot contact on the ground between the two feet; we defined foot contact as the local minima of the mid-foot marker vertical velocity profile [34]. We then determined step-width variability as the standard deviation across three consecutive step widths in a given trial.

We used JMP 14 software (SAS Institute Inc., Cary, NC) with an alpha level of 0.05 for all statistical analyses. Data for each measure met the assumptions of an ANOVA, that is, the model residuals were normally and independently distributed with zero mean and constant variance [35]. For two of our measures, we noted a potential outlier based on the distribution of studentized residuals. Thus, we ran the ANOVA for these measures with and without the outlier (see Results for details). For each task, we were interested in determining the effect of stimulation condition (SVS or sham), balance condition (challenged or unchallenged), and threshold technique (sinusoidal-based or cutaneous-based) on our measures. Thus, we used separate three-way (stimulation condition x balance condition x threshold technique) mixed model ANOVAs for each measure. We included participant as a random effect, and used Tukey’s post hoc tests when we found a significant interaction. All data used to produce statistical results and figures are available at https://osf.io/dtmaq.

## Results

### Effects of SVS on stability during standing

**Fig 2:**
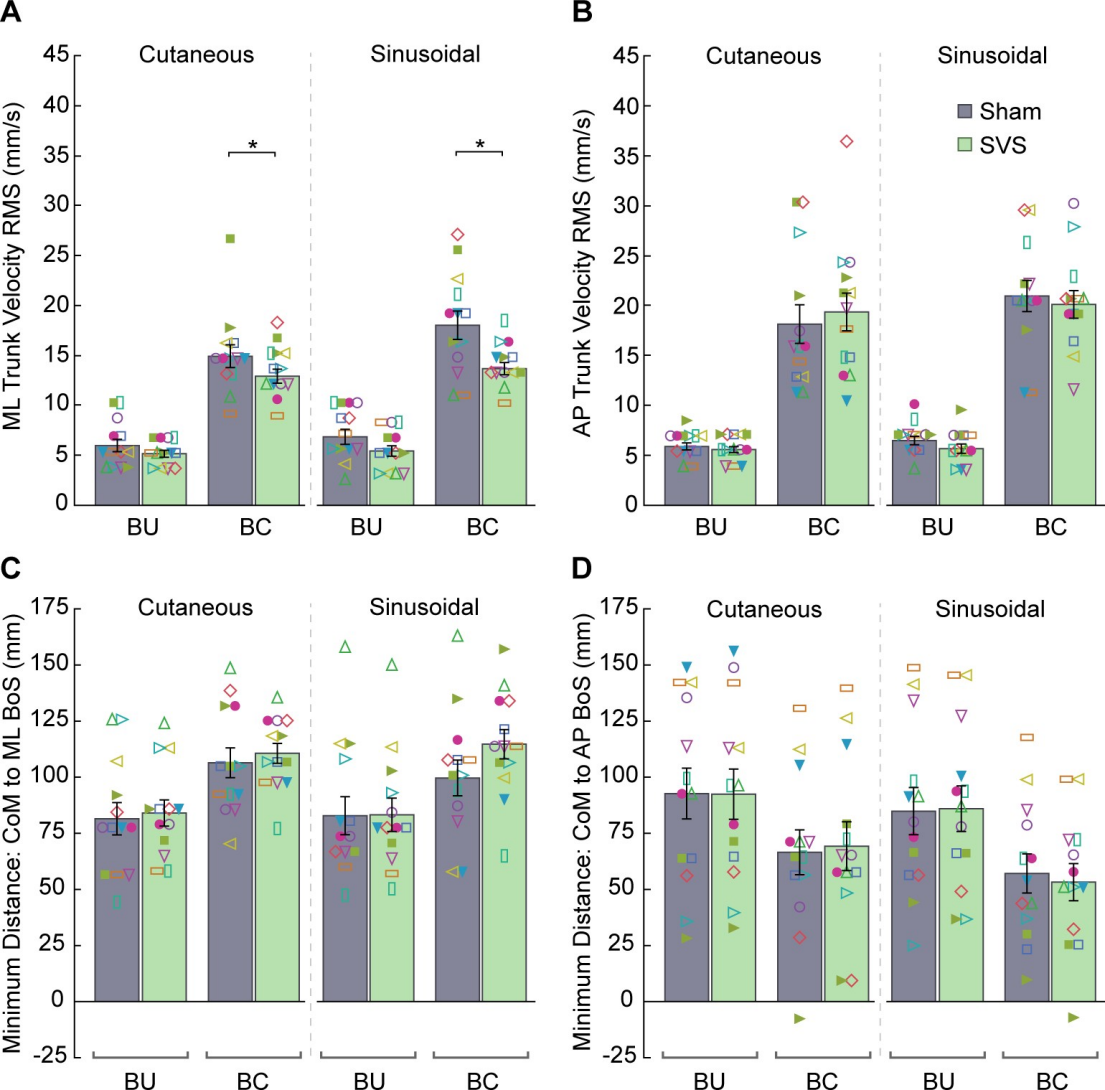
Results for the standing tasks. (A) Medial-lateral (ML) trunk velocity root mean square (RMS). (B) Anterior-posterior (AP) trunk velocity RMS. (C) The minimum distance between the center of mass (CoM), based on a chest position marker, and the ML base of support (BoS) during the standing task. (D) The minimum distance between the CoM and the AP BoS during the standing task. Data are mean ± SE. Individual participant data are superimposed. Asterisks indicate differences between sham and stochastic vestibular stimulation (SVS) conditions based on post-hoc tests following a significant balance condition x stimulation condition interaction (p < 0.05). Details of the other significant effects are reported in Table 2.

Eleven out of thirteen participants lost their balance (engaged the safety harness, took a step, or required experimenter assistance) at least once while standing on the rubber hemispheres during the balance-challenged condition. None of the participants lost their balance during the normal, balance-unchallenged condition, as to be expected. These results, in conjunction with our measures of stability (see Fig 2 and Table 2), strongly support the notion that the rubber hemispheres challenged balance while standing.

**Table 2:** Results of three-way ANOVAs for the standing tasks.

**Measure**–main effects	**Threshold Technique**	**Balance Condition**	**Stimulation Condition**	
ML Trunk Velocity RMS	F_1,84_ = 6.8,	F_1,84_ = 351.8,	F_1,84_ = 19.9,	
	p = 0.011**	p < 0.0001*	p < 0.0001*	
AP Trunk Velocity RMS	F_1,84_ = 1.9,	F_1,84_ = 311.7,	F_1,84_ = 0.06,	
	p = 0.177	p < 0.0001*	p = 0.808	
Min. Distance: CoM to ML BoS	F_1,84_ = 0.03,	F_1,84_ = 63.1,	F_1,84_ = 3.1,	
	p = 0.864	p < 0.0001*	p = 0.081	
Min. Distance: CoM to AP BoS	F_1,84_ = 9.8,	F_1,84_ = 74.5,	F_1,84_ = 0.001,	
	p = 0.002*	p < 0.0001**	p = 0.981	
**Measure–**interactions	**Threshold x Balance**	**Threshold x Stimulation**	**Balance x Stimulation**	**Threshold x Balance x Stimulation**
ML Trunk Velocity RMS	F_1,84_ = 2.0,	F_1,84_ = 2.4,	F_1,84_ = 4.5,	F_1,84_ = 0.9,
	p = 0.164	p = 0.128	p = 0.037***	p = 0.356
AP Trunk Velocity RMS	F_1,84_ = 0.9,	F_1,84_ = 0.7,	F_1,84_ = 0.2,	F_1,84_ = 0.3,
	p = 0.359	p = 0.415	p = 0.633	p = 0.618
Min. Distance: CoM to ML BoS	F_1,84_ = 0.07,	F_1,84_ = 0.5,	F_1,84_ = 1.7,	F_1,84_ = 1.1,
	p = 0.798	p = 0.493	p = 0.196	p = 0.304
Min. Distance: CoM to AP BoS	F_1,84_ = 0.8,	F_1,84_ = 0.2,	F_1,84_ = 0.02,	F_1,84_ = 0.4,
	p = 0.382	p = 0.681	p = 0.882	p = 0.532

ML = medial-lateral; AP = anterior-posterior; RMS = root mean square; CoM = center of mass; BoS = base of support; SVS = stochastic vestibular stimulation.

* P < 0.05: cutaneous > sinusoidal or balance-challenged > balance-unchallenged or sham > SVS.

** P < 0.05: sinusoidal > cutaneous or balance-unchallenged > balance-challenged.

*** P < 0.05; this significance disappears when a single outlier is removed (see text for details).

To determine the effect of SVS on standing, we first analyzed ML and AP trunk velocity RMS. ML trunk velocity RMS differed depending on the balance condition and presence of SVS (balance x stimulation condition interaction: F_1,84_ = 4.5, p = 0.037). Post-hoc tests revealed a reduction in RMS with SVS in the balance-challenged, but not the balance-unchallenged condition (Fig 2A). In fact, in the balance-challenged condition, we found a 24% decrease in ML trunk velocity RMS with SVS compared to sham when the mean data from both cutaneous and sinusoidal conditions were combined. The mixed model analysis revealed a potential single outlier in this measure’s data (i.e., studentized residual of approximately 4). Excluding this outlier altered the p-values of the fixed effect tests such that we no longer had a significant balance x stimulation condition interaction (p = 0.071). Regardless, statistically significant main effects of threshold technique, balance condition, and stimulation condition remained after removing the outlier. In addition, pairwise Tukey post-hoc comparisons continued to show a significant reduction in ML trunk velocity RMS with SVS in the balance-challenged, but not the balance-unchallenged condition. Since we found nothing overtly wrong with the data from the outlier and pairwise post-hoc comparisons were identical, we chose to keep the outlier in the model (and statistical test results with the outlier in the model are reported in Table 2). Although ML trunk velocity RMS was greater in the sinusoidal compared to the cutaneous condition, as reflected by a main effect of threshold technique (F_1,84_ = 6.8, p = 0.011), we did not find any significant interaction of the stimulation intensity with other factors (Table 2).

For AP trunk velocity RMS (Fig 2B), we found no significant effect of stimulation (Table 2). However, we found a 232% increase in AP trunk velocity RMS in the balance-challenged condition compared to the balance-unchallenged condition (F_1,84_ = 311.7, p < 0.0001). The mixed model analysis revealed a potential single outlier in this measure’s data as well (i.e., studentized residual of approximately 4). Excluding this value altered the p-values of the fixed effect tests but did not lead to any change in significance. Statistical test results in Table 2 are for the model with the outlier included.

Next, we compared the minimum distance from the trunk to the ML and AP edges of the base of support to determine the effect of SVS on stability. Here, larger values suggest greater stability, as the CoM is further from the edge of the base of support. Participants demonstrated 30% greater minimum ML distance in the balance-challenged condition compared to the balance-unchallenged condition (Fig 2C; F_1,84_ = 63.1, p < 0.0001). This larger value is likely a reflection of the increased ML distance between the participant’s feet in the balance-challenged condition due to the rubber hemispheres. In contrast, participants demonstrated a 31% decrease in the minimum AP distance in the balance-challenged versus balance-unchallenged condition (Fig 2D; F_1,84_ = 74.5, p < 0.0001). In addition, we found a smaller minimum AP distance for the sinusoidal condition compared to the cutaneous condition (F_1,84_ = 9.8, p = 0.002). The remaining ANOVA results for each measure are reported in Table 2, which show no significant effects of SVS.

### Effects of SVS on overground walking

**Fig 3:**
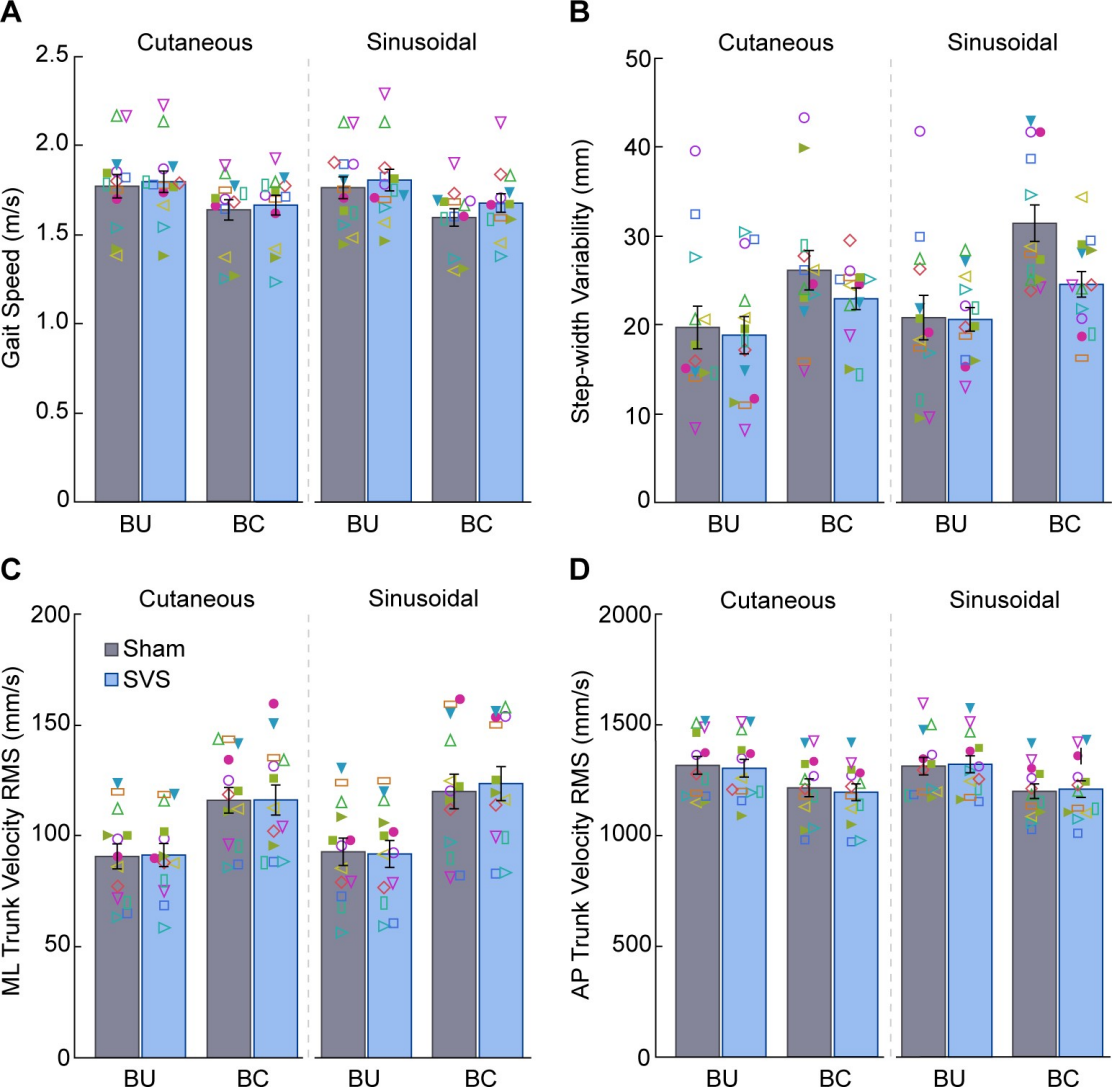
Results for the walking tasks. (A) Gait speed. (B) Step-width variability. (C) Medial-lateral (ML) trunk velocity root mean square (RMS). (D) Anterior-posterior (AP) trunk velocity RMS. Data are mean ± SE. Individual participant data are superimposed. Gait speed increased and step-width variability decreased with SVS (stimulation condition main effect, p < 0.05). Details of the main effects of threshold technique, balance condition, and stimulation condition are reported in Table 3.

The balance-challenged condition affected walking in several ways. As illustrated in Fig 3A, participants walked significantly slower in this condition (F_1,84_ = 77.9, p < 0.0001). As illustrated in Fig 3B, participants had 31% greater step-width variability in the balance-challenged condition compared to the balance-unchallenged condition (F_1,84_ = 30.6, p < 0.0001). We also found differences in ML (Fig 3C) and AP (Fig 3D) trunk velocity RMS. Specifically, participants demonstrated 30% greater ML trunk velocity RMS in the balance-challenged condition compared to the balance-unchallenged condition (F_1,84_ = 260.2, p < 0.0001). Interestingly, however, participants exhibited 8% less AP trunk velocity RMS in the balance-challenged condition versus the balance-unchallenged condition (F_1,84_ = 137.0, p < 0.0001).

SVS led to significant changes in walking performance. Specifically, participants walked 3% faster with SVS compared to sham stimulation regardless of condition (Fig 3A), as shown by a main effect of stimulation (F_1,84_ = 7.2, p = 0.009). Furthermore, participants demonstrated an 11% reduction in step-width variability with SVS compared to sham stimulation regardless of condition (Fig 3B), as shown by a main effect of stimulation (F_1,84_ = 6.1, p = 0.016). However, SVS had no effect on ML (Fig 3C) and AP (Fig 3D) trunk velocity RMS (p > 0.05). The full results of the ANOVAs for each measure are reported in Table 3.

**Table 3:** Results of three-way ANOVAs for the walking tasks.

**Measure**–main effects	**Threshold Technique**	**Balance Condition**	**Stimulation Condition**	
Gait speed	F_1,84_ = 0.1,	F_1,84_ = 77.9,	F_1,84_ = 7.2,	
	p = 0.746	p < 0.0001**	p = 0.009***	
Step-width variability	F_1,84_ = 4.6,	F_1,84_ = 30.6,	F_1,84_ = 6.1,	
	p = 0.034*	p < 0.0001*	p = 0.016*	
ML Trunk Velocity RMS	F_1,84_ = 4.1,	F_1,84_ = 260.2,	F_1,84_ = 0.3,	
	p = 0.045*	p < 0.0001*	p = 0.615	
AP Trunk Velocity RMS	F_1,84_ = 0.1,	F_1,84_ = 137.0,	F_1,84_ = 0.2,	
	p = 0.730	p < 0.0001**	p = 0.682	
**Measure–**interactions	**Threshold x Balance**	**Threshold x Stimulation**	**Balance x Stimulation**	**Threshold x Balance x Stimulation**
Gait speed	F_1,84_ = 0.2,	F_1,84_ = 1.3,	F_1,84_ = 0.5,	F_1,84_ = 0.3,
	p = 0.695	p = 0.266	p = 0.480	p = 0.590
Step-width variability	F_1,84_ = 0.8,	F_1,84_ = 0.4,	F_1,84_ = 3.9,	F_1,84_ = 0.9,
	p = 0.380	p = 0.511	p = 0.051	p = 0.345
ML Trunk Velocity RMS	F_1,84_ = 1.7,	F_1,84_ = 0.06,	F_1,84_ = 0.3,	F_1,84_ = 0.6,
	p = 0.202	p = 0.808	p = 0.557	p = 0.450
AP Trunk Velocity RMS	F_1,84_ = 0.2,	F_1,84_ = 1.9,	F_1,84_ = 0.01,	F_1,84_ = 0.03,
	p = 0.645	p = 0.174	p = 0.906	p = 0.860

ML = medial-lateral; AP = anterior-posterior; RMS = root mean square; SVS = stochastic vestibular stimulation.

* P < 0.05: balance-challenged > balance-unchallenged or SVS < sham or sinusoidal > cutaneous.

** P < 0.05: balance-unchallenged > balance-challenged.

*** P < 0.05: SVS > sham.

## Discussion

Previous research has shown the benefits of SVS in certain standing and walking situations. Here, we had healthy adults standing and walking under sustained balance-unchallenged and balance-challenged conditions. The inflated rubber hemispheres clearly challenged balance; for example, we found greater ML trunk velocity RMS during both standing and walking. Importantly, we show that when standing balance is challenged, and the eyes are open, subthreshold SVS reduces ML trunk velocity RMS. Regardless of condition, we also show that subthreshold SVS decreases step-width variability and marginally increases gait speed when walking with the eyes open. We found similar effects in both tasks regardless of whether the SVS intensity was based on cutaneous or sinusoidal threshold techniques. Taken together, our results extend our understanding of the positive effects of SVS to balance-challenged, eyes-open standing and walking.

SVS significantly reduced ML trunk velocity RMS under balance-challenged, but not balanced-unchallenged, quiet standing. The fact that standing on normal ground with the eyes open is not overly challenging likely explains the lack of SVS effect in our balance-unchallenged condition. Recently, several research groups have found that, in healthy young adults, SVS leads to improved standing balance under normal support surface conditions [16,17] as well as while standing on foam [21,22] with the eyes closed. Most studies of this nature have participants standing for between 30 to 60 seconds in duration while SVS is applied. Despite using a much shorter stimulation and trial duration—because of the difficulty of standing on the rubber hemispheres in the balance-challenged condition—we still found effects of SVS. This supports previous work that used only five seconds of SVS during standing [16]. It is possible that we would have observed even greater effects of SVS with longer standing and stimulation durations. In contrast to our study, Pal et al. [23] reported no significant effect of SVS on postural sway among healthy adults with the eyes open and standing on foam. Apart from challenging balance in a different manner, the major difference between our studies is that Pal et al. [23] used the same set of stimulation intensities for all participants. Based on our results, and the results of others, we argue that finding a participant’s optimal stimulation intensity (using a common threshold technique) is critical for demonstrating the effectiveness of SVS.

SVS significantly decreased step-width variability and marginally increased gait speed regardless of walking condition. This supports other studies that reported walking benefits—including faster gait speed and improved stride measures—observed in healthy adults [18,20,24] and bilateral vestibulopathy patients [18,19]. Only one other study in addition to ours has investigated how SVS alters measures of balance during a balance-challenged walking task. Specifically, Mulavara et al. [24], using a sinusoidal support surface perturbation while participants walked on a treadmill, reported a reduction in the variability of gait cycle timing and ML linear trunk acceleration with SVS. However, to the best of our knowledge, we are the first to show how SVS affects balance-challenged overground walking. Thus, given known differences between treadmill and overground walking [25–27], it appears that the effectiveness of SVS is relatively robust between different walking tasks and balance challenges. Although people do not typically walk with rubber inflatable hemispheres under their shoes, this balance manipulation may resemble walking across unstable terrain, including terrain with some degree of compliance, like soggy grass, mud, or snow. Testing how SVS affects balance when walking across this type of terrain may be a worthwhile future avenue of research.

There are several potential limitations with our walking task that may have minimized the effects of SVS. First, we only analyzed data across three steps in the middle of an ~6 m walkway due to limited motion capture volume, as opposed to a greater number of steps (or longer distance) used in previous studies (e.g., [18,20]). Second, our participants walked at a self-selected speed that was faster than the majority of studies. Vestibular involvement in gait is stronger with slower walking (e.g., [36]) and SVS may lead to greater effects with slower speeds [20], though see Mulavara et al. [24], who show SVS benefits with gait speeds closer to ours. Third, the vestibular system may play a smaller role when walking with the eyes open, as in our experiment. However, eyes-open walking is more realistic, and arguably more clinically relevant; it is encouraging that we observed improvements despite this manipulation. Fourth, although we tested the effects of SVS using amplitudes derived from two different (yet common) threshold methods, it is possible that optimal intensities differ between standing and walking [17,18]. Fifth, SVS may not be powerful enough to fully overcome such a large amount of instability created by the inflatable rubber hemispheres. And sixth, it is also possible that the benefits of SVS in overground walking are more prevalent in neurological populations who have greater capacity for improvement.

What neural mechanisms may be responsible for the improvements in standing and walking observed in our study? Electrical vestibular stimulation activates the afferents of both semicircular canals and otolith organs, rather than the end organs themselves, and the brain appears to process the net sum of this activation through an internal model of Earth’s gravity [30–32,37]. SVS may improve sensory perception [6]. Consequently, SVS may facilitate the detection of head acceleration, thus allowing for greater balance control through activation of the vestibulospinal tract and its indirect connections to anti-gravity muscles of the legs [38]. Alternatively, or in conjunction, SVS may affect downstream brain regions via the vestibular nuclei. The vestibular nuclei project to the cerebellum, a region that receives limb and body proprioceptive signals [38] and is important for balance [39]. The vestibular nuclei also receive input from the cerebellum, as well as neck and limb somatosensory feedback [38,40,41]. Given the known roles of cutaneous [42] and ankle proprioception [43,44] in balance control, these nuclei can thus integrate a variety of input to facilitate balance. Vestibular stimulation also affects a broad range of cortical and subcortical areas, including frontal, parietal, temporal, and insular cortices, as well as thalamus, basal ganglia, and cerebellum [45–47]. This is significant given the recent appreciation of the cortical contribution to standing balance [48–50] and walking [51,52]. Interestingly, electroencephalography-based studies report increased theta power and individual alpha peak frequency in frontal, central, and parietal cortical areas with greater balance challenge [48,49]. Ultimately, the exact effects of SVS on brain networks remain unclear.

The choice of threshold technique to determine the stimulation amplitude did not greatly affect our results despite the fact that they produced different amplitudes. Recent studies in standing [21,22] and walking [20] that used either one of these techniques demonstrated significant improvements in balance, thus supporting our findings. Some groups have also shown that there are optimal stimulation amplitudes [17,18,22,24], which may differ depending on the task; the values from these studies match ours relatively well. Overall, our results suggest that it does not seem to matter whether a cutaneous or sinusoidal threshold technique is used to determine stimulus amplitude, though this is predicated on using recommended threshold percentages (i.e., 50% of sinusoidal motion threshold and 80% of cutaneous threshold). However, amplitude is just one parameter of the stimulation signal to consider. Different groups have used frequency ranges of 0.02 to 10 Hz [13,18], 0.1 to 10 Hz [46], 0 to 30 Hz [20–22,24], which is similar to the present study, and even 0.1 to 640 Hz [16]. At this point, the optimal stimulation frequency range is unknown. Given the diversity of stimulation frequencies and intensities in the literature, and the finding that individuals have different optimal intensities [17,18,22,24], it will be important to determine how different parameters dictate the effectiveness of SVS in both healthy and neurological populations.

In conclusion, we found performance improvements in eyes-open standing balance and walking with SVS. Both sinusoidal and cutaneous threshold techniques produced similar effects. Future research should investigate whether there is a graded effect of SVS based on how much balance is challenged. Furthermore, this research should also determine whether different frequency ranges are more effective at reducing instability during both standing and walking.
